# The use of interactive and engaging events in Austin, Texas, to promote the US return of IQOS

**DOI:** 10.18332/tpc/214726

**Published:** 2026-03-28

**Authors:** Josephine T. Hinds, Eugene M. Talbot, Ollie Ganz, Cristine D. Delnevo

**Affiliations:** 1 Department of Psychological, Health, and Learning Sciences, The University of Houston, Houston, United States; 2 Rutgers Institute for Nicotine and Tobacco Studies, Rutgers Health, New Brunswick, United States; 3 Department of Health Behavior, Society and Policy, Rutgers School of Public Health, Piscataway, United States

**Keywords:** IQOS, heat-not-burn, tobacco industry

## Abstract

IQOS is a heated tobacco product from Philip Morris International (PMI) with modified risk tobacco product (MRTP) designation from the U.S. Food and Drug Administration (FDA). The commercial relaunch of IQOS featured experiential marketing centered in Austin, Texas, and began in October 2024. Invitation-only events were offered to adults aged ≥21 years who resided near Austin/Travis County and confirmed they were tobacco users when they joined the 'IQOS Circle'. Events were free-of-charge and included live music concerts, movie premieres, and seasonally and locally themed parties. Events offered free food, drinks, and giveaways, and usually included PMI representatives who invited discussion and questions about IQOS, where customers could hold devices with the promise that opportunities to try IQOS were coming soon. A two-day downtown block party marked IQOS’ commercial release in March 2025, offering 'guided experiences' to customers who could then initiate a 14-day trial. The IQOS device and charging case with ten packs of HEETS sticks cost $1.00, plus a non-refundable $40 'trial fee'. Within the trial window, customers could return the product free of charge or pay an additional $60.00 to keep the device. In April 2025, IQOS products were made available for purchase online to Austin-area zip codes, and on 2 September 2025, shipping expanded to 'selected major cities' in Texas, including Dallas, Houston, and San Antonio. Events in Austin, TX and subsequent pilots in Ft. Lauderdale, FL, and Jackson, MS will inform PMI’s launch of ILUMA, their most advanced IQOS model currently awaiting FDA authorization. It remains to be seen whether IQOS will provide health benefits to individuals who switch completely to IQOS from cigarettes, or whether those initiating IQOS are cigarette-naïve and/or otherwise not using nicotine products. Continued monitoring of the use and appeal of IQOS will be essential to tobacco control and public health efforts.

## INTRODUCTION

IQOS is a heated tobacco product from Philip Morris International (PMI), with two models (IQOS 2.4 and IQOS 3) and HEETS sticks are available in select US markets as of August 2025. IQOS appeared in the US in 2019^1^, after receiving marketing authorization from the U.S. Food and Drug Administration (FDA) via the premarket tobacco product application (PMTA) pathway^2^. The FDA later granted IQOS modified risk tobacco product (MRTP) authorization in July 2020, which allows PMI to make certain reduced exposure claims about IQOS (e.g. ‘Scientific studies have shown that switching completely from conventional cigarettes to the IQOS system significantly reduces your body’s exposure to harmful or potentially harmful chemicals’)^3^. In September 2021, the US International Trade Commission (ITC) issued cease-and-desist orders banning US sales of IQOS due to a patent infringement case filed by Reynolds American (subsidiary of British American Tobacco [BAT]). The ban was lifted in May 2024 after PMI and BAT reached a settlement. Shortly thereafter, PMI heralded the relaunch of IQOS in the US using online advertising, branded social media, and experiential marketing (i.e. encouraging consumer engagement at recreational venues and events)^4^. The re-launch centered in Austin, Texas, a tech-forward city populated by young professionals with a prominent art and music culture. This article describes the experiential marketing integral to the promotion of IQOS in Austin.

### Brand-building and promotional events

Beginning in October 2024, the US-based IQOS website^5^ and official social media accounts promoted the ‘IQOS Circle’ as a chance to ‘Be the First’ to try IQOS. Website access and Circle membership were restricted to age-verified adults ≥21 years residing near Austin/Travis County who confirm they use tobacco products. Circle members received text message invitations to exclusive, free events that included food, drinks, and giveaways (e.g. custom bracelets from a local jeweler).

**Figure 1:**
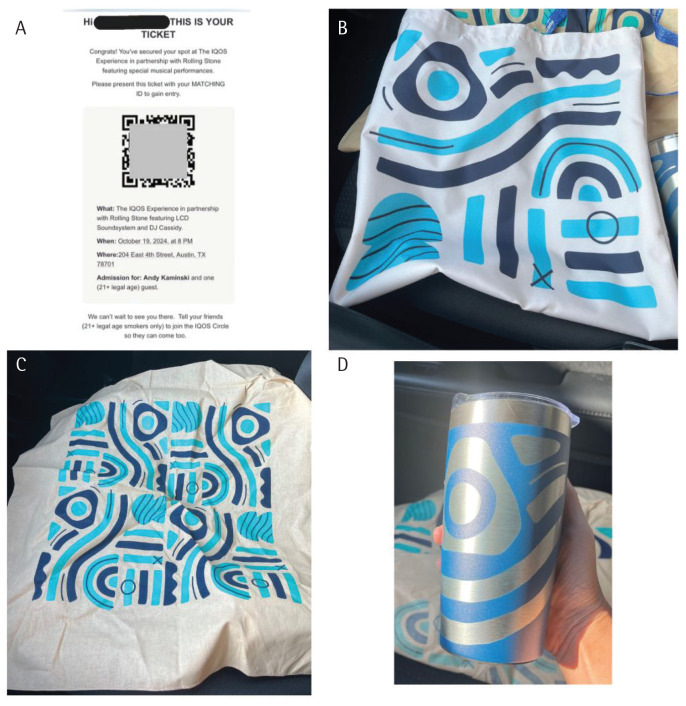
Promotional items from October 2024 event in Austin, Texas. Shown clockwise from top left: A copy of the virtual ticket to the event, a tote bag, an insulated drink tumbler with lid, a bandana

The first promotional event occurred on 19 October 2024: ‘The IQOS Experience in partnership with Rolling Stone featuring LCD Soundsystem and DJ Cassidy’. Using ‘mystery marketing’ techniques, an Austin-focused Instagram account^6^ posted a since-deleted, cryptic message about an upcoming ‘pop-up’ show by a Grammy Award-winning artist, directing viewers to the IQOS Circle website to join and claim two free tickets. Attendees entered the venue in small groups, observing an IQOS promotional pitch from PMI representatives before entry. No IQOS branding presence existed within the venue, although there were free promotional items (a bandana, a tote bag, and an insulated tumbler) in IQOS colors (Figure 1A-1D). Later events included more explicit promotional branding, including display cases of IQOS devices with PMI representatives inviting discussion and questions. Examples of promotional events included a premiere of the documentary ‘Row of Life’ (with free passes for local canoe/kayak rental), seasonally themed parties (e.g. an ‘après ski’ holiday party in December 2024), and Austin-themed local events (e.g. live music and tequila tasting during a custom leatherwork/stamping clinic). At each event and on social media, representatives promised that IQOS would be ‘coming to Austin soon’, though attendees could not yet use or purchase the product at events. Additionally, two standing downtown Austin ‘pop-ups’ offered a ‘sneak peek of the product and chat with IQOS experts’, described as a ‘look and touch’ experience.

### Commercial release

**Figure 2:**
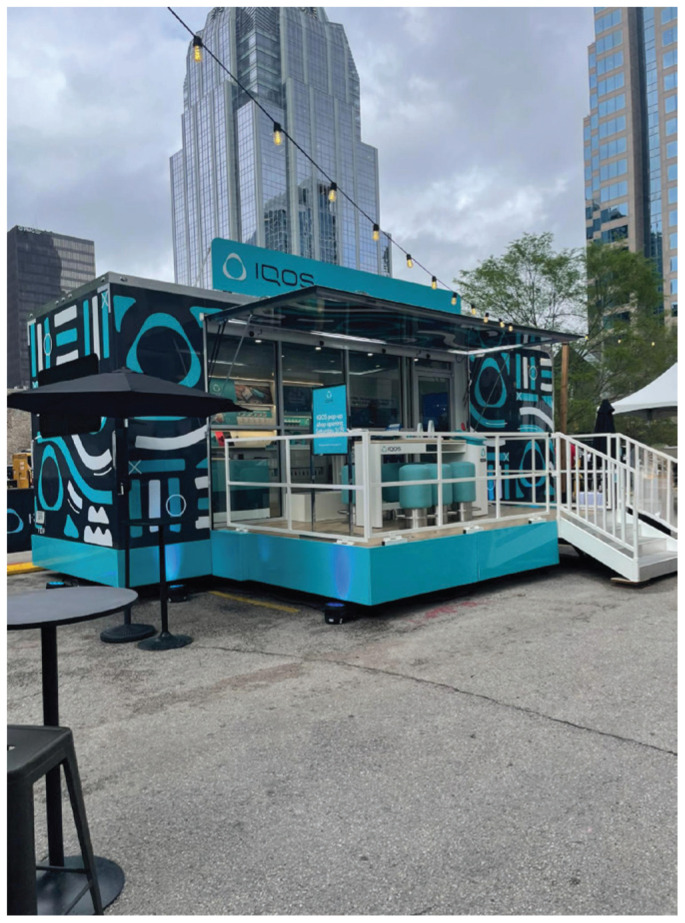
Guided experience trailer (outside) at commercial launch block party, Austin, Texas, 28 March 2025. An IQOS trailer used to host guided experiences

**Figure 3:**
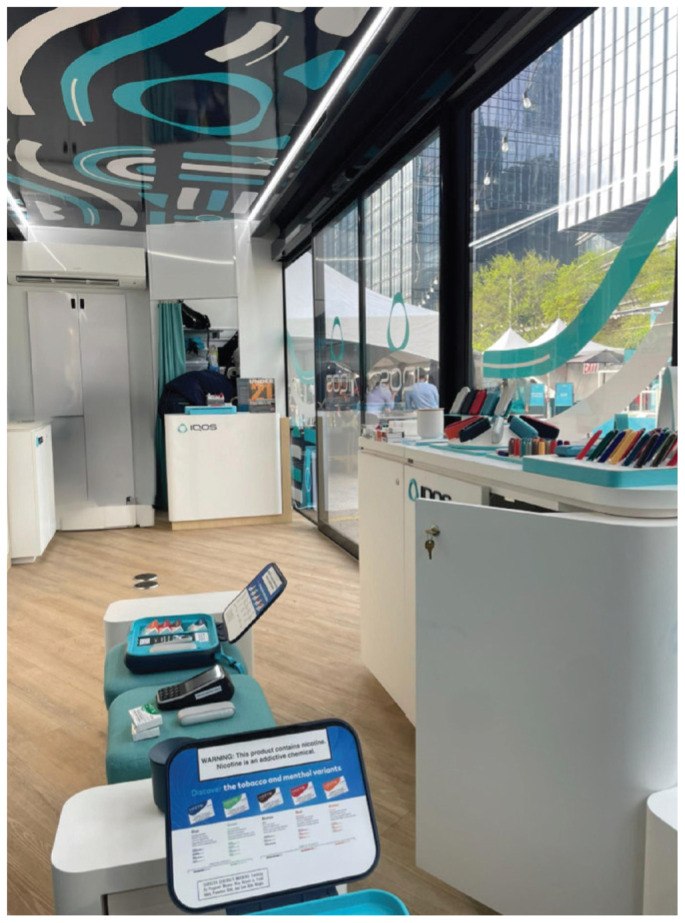
Guided experience trailer (inside) at commercial launch block party, Austin, Texas, 28 March 2025. A view from inside the guided experience trailer

**Figure 4:**
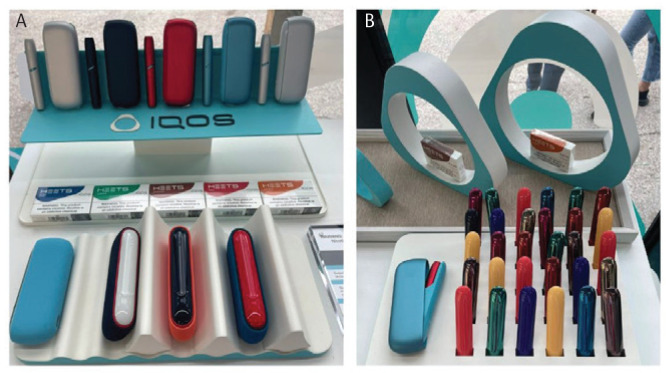
IQOS display cases inside guided experience trailer at commercial launch block party, Austin, Texas, 28 March 2025. Displays with customizable color options for charging case and IQOS devices

**Figure 5:**
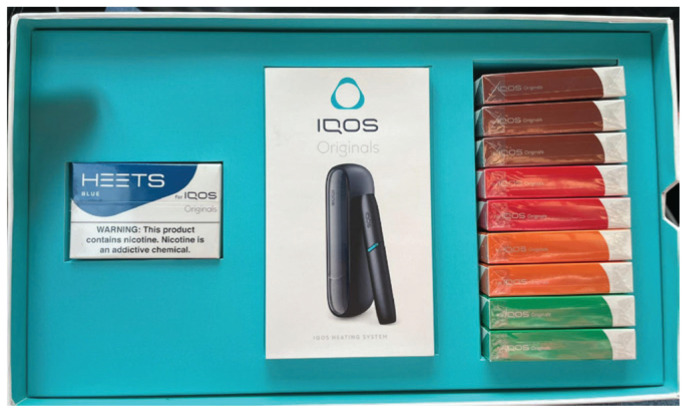
IQOS starter kit from commercial launch in Austin, Texas, 28 March 2025. A photograph of the IQOS starter kit, purchased at the commercial launch event in March 2025 in Austin. The kit contained the IQOS Originals heating system (HEETS stick holder with pocket charging case) and ten packages of HEETS sticks

A two-day launch event marked IQOS’ commercial release. During 28–29 March 2025, Circle members attended an invitation-only downtown block party featuring food trucks, bar tents, a music stage, and multiple air-conditioned trailers and tents where IQOS representatives hosted ‘guided experiences’ (Figures 2–5).

‘Experiences’ included one-on-one demonstrations of how to use and maintain the IQOS device, and customers could try any of the five available HEETS varieties: blue, green, bronze, red, and amber. Customers were subsequently invited to a 14-day trial, which entailed purchasing an IQOS HEETS stick holder with pocket charging case and ten packs of HEETS sticks for $1.00, plus a non-refundable $40 ‘trial fee’. Within the 14-day window, customers could return the product free-of-charge, or pay an additional $60.00 to keep the device. Customers received frequent check-in text messages from ‘IQOS Care Experts’ throughout the trial offering guidance and encouragement in using IQOS. Attendees to the launch were also gifted a one-day pass to that weekend’s MotoGP Red Bull Grand Prix of the Americas in Austin, where a promotional trailer in the ‘Fan Zone’ offered similar activities as the downtown event.

In April 2025, the online retail shop made IQOS devices, chargers, HEETS, and accessories available for purchase after location and ID-verification, limited to Austin-area zip codes. On 2 September 2025, IQOS announced expanded shipping to ‘select major cities’ in Texas, including Dallas, Houston, and San Antonio.

## COMMENTARY

IQOS has met considerable commercial success globally^7,8^, but how it will fare in the US remains unknown. In discussing 2024 third-quarter results, PMI leadership shared that pilots in Austin and other cities would inform their approach for the at-scale launch of ILUMA, their most advanced model currently awaiting FDA authorization^9^. In May 2025, the brand announced expansion into Ft. Lauderdale, Florida via Instagram. An ‘IQOS Discovery Event’ appeared similar to Austin’s, conducted during the Formula 1 Crypto.com Miami Grand Prix weekend, perhaps signaling their continued experiential marketing.

The marketing strategies used in Austin appear specifically tailored to its young/hip demographic. Austin’s median age is the sixth-lowest among the 50 largest US metropolitan areas^10^, and fewer than ten percent of 18–29-year-olds in Travis County reported current cigarette smoking in 2022^11^, likely due in part to Austin’s 2005 citywide public smoking ban, including bars, restaurants, and music venues. Experiential marketing is a tried and true tactic for tobacco companies who understand the importance of young adult activity and venues (e.g. bars) when promoting cigarettes to young adults^12-14^. The intention may be targeting young adults specifically due to the fact that young adulthood is characterized by experimentation with addictive substances^15^. Indeed, 11.2% of Travis County young adults currently used e-cigarettes in 2022^11^, and it is unclear how many of them concurrently used cigarettes.

## CONCLUSION

It remains to be seen whether IQOS will provide benefits to individuals who smoke cigarettes by switching completely to IQOS, which FDA has authorized for sale as a modified risk tobacco product^3^, or whether those initiating IQOS are cigarette-naïve and/or otherwise not using nicotine products. Continued monitoring of the use and appeal of IQOS will be essential to tobacco control and public health efforts.

## Data Availability

Data sharing is not applicable to this article as no new data were created.
